# Supplementary material for: Magnetic and electric field accelerate Phytoextraction of copper Lemna minor duckweed
Source: PLoS One. 2021 Aug 4;16(8):e0255512. doi: 10.1371/journal.pone.0255512 (PMC8336833; doi:10.1371/journal.pone.0255512)
Supplement: Attachment — Submitted filename: Comments - Manuscript 23_03_21 Politaeva Badenko_PlosOne_ver2.docx [file pone.0255512.s001.docx]

**Magnetic and Electric Fields Accelerate Phytoextraction of Copper through Lemna Minor Duckweed**

**Natalia Politaeva^1^, Vladimir Badenko^1*^**

*^1^ Civil Engineering Institute, Peter the Great Saint Petersburg Polytechnic University, Saint Petersburg, Politechnicheskaya st., 29, Russian Federation*

*^*^ Corresponding author:* badenko_vl@spbstu.ru

**Abstract**

The international agencies such as the World Health Organization and the World Water Council have identified a crisis in water supply and an escalation of problems associated with fresh water. The urgent task is to develop effective technologies for wastewater treatment for their discharge into water bodies or reuse according to principals of cleaner production resources and their pollution. Phytoremediation biotechnologie is the most environment friendly and cheapest way to clean water and suitable for ongoing environmental restriction and sustainable developments. The main disadvantage of phytoremediation process is the slow pace. The phytoremediation acceleration method proposed in this paper is to analyze the use of biosorbents for wastewater treatment. The objective of this research is to study the influence of the magnetic field strength, and the combined effects of magnetic and weak electric field on the process of copper extraction using the higher aquatic plant *Lemna minor*. The results of research on method for accelerating phytoremediation for purifying wastewater from copper ions presented in this paper are relevant in context of sustainable development and cleaner production. The method proposed became a basis for the original scheme for purifying wastewater from copper with the subsequent release of copper from the spent phytomass. Technological recommendations have been developed for the use of this phytoremediation processes according to the principle of bioeconomics. This scheme makes it possible to achieve waste-free technologies and a closed cycle of duckweed using. The method and technology proposed has a number of advantages: high purification efficiency, low cost and low energy consumption, versatility, environmental safety, ease of implementation, and wastelessness.

***Key words:*** *wastewater treatment, phytoremediation, duckweed, copper cations, magnetic field influence, weak electric field influence, technological recommendations.*

1. **Introduction**

The growth of the world's population, industrialization, urbanization and the intensification of agricultural production have led to a crisis in water supply and an escalation of problems associated with fresh water resources and their pollution, especially in developing countries [1, 2]. Clean water demand and the number of people experiencing a scarcity of water resources is constantly growing. This problem is pointed out by international agencies such as the World Health Organization (WHO) and the World Water Council [3, 4]. In this connection, the urgent task is to develop effective technologies for wastewater (WW) treatment for their discharge into water bodies or reuse according principals of cleaner production [2, 5, 6].

Heavy metal ions (HMI), which are usually present in industrial and agricultural WW, are especially toxic [7]. Heavy metals (such as Pb, Zn, Hg, Cd, Cr, As, Cu, Mn, Fe, Ni, Co) entering a human body can cause serious diseases and have the property of accumulating in the food chain, are resistant to biodegradation and are usually stable in the environment [8, 9]. However, on the other hand, heavy metals such as copper, zinc, boron, molybdenum are necessary for the growth and development of animals and plants, but they are harmful when their concentrations exceed permissible limits [10]. Therefore, various regulatory agencies, both global – WHO and national (US EPA - United States Environmental Protection Agency) level install regulation for the acceptable limit of heavy metals in drinking water to rule out possible health problems [11-13].

Urban, agrarian and industrial WW treatment technologies can be classified into physical, chemical and biological methods, each of which have its own advantages and disadvantages [14]. Heavy metals are removed from WW by methods such as membrane filtration, chemical precipitation, adsorption, chelation, and ion exchange [15-18]. It should also be noted that heavy metals are widely used in modern technologies. Therefore, their recovery from WW for reuse can bring not only environmental, but also economic benefits, which is the way to a circular economy and sustainable development [19-20]. The analysis shows that despite recent advances in water purification (ion exchange, photocatalysis and adsorption processes), most existing methods for water treatment have high operating costs and limitations in terms of the efficiency of removing contaminants [21]. For example, the adsorbents used in these technologies are relatively expensive and therefore not always available in developing countries, where the problem of clean drinking water is especially acute [1, 22, 23].

Compared to physical and chemical technologies, biotechnology costs are lower for WW treatment in real situation and more suitable for ongoing environmental restriction and sustainable developments [24]. Recently, especially in warm countries, the technology of purification of WW has been intensively introduced, with the help of higher aquatic plants well known as phytoremediation [25-27]. The plant cell is able to efficiently extract and utilize pollutants and no by-products accumulate. Phytoremediation is the most environmentally friendly and cheapest way to clean water [28-30]. The dead and depleted plants – phytosorbents – are non-toxic and can be used as additives to animal and bird feeds to produce compost, vermicompost and biodiesel [31, 32].

The term *phytoremediation* (*phyto* - plant and *remediation* - redress) has a not very long history. It was first used by the American scientist I. Raskin in 1994 [33]. Higher aquatic plants in reservoirs perform the following main functions, which are of the greatest importance for environmental sustainability: *filtration* (contribute to sedimentation of suspended solids); *absorbing* (absorption of nutrients and some organic substances); *cumulative* (the ability to accumulate some metals and organic substances that are difficult to decompose); *oxidative* (in the process of photosynthesis, water is enriched with oxygen); *detoxification* (plants are able to accumulate toxic substances and convert them to non-toxic) [29].

The expansion of the use of phytoremediation technologies is observed in all countries of the world [27, 34]. For example, structures with wetland vegetation for treatment of domestic WW in Ireland [35], the Netherlands [36], China [37] are described. In China, phytoremediation technologies are used for both soil and polluted water treatment [24, 27, 38]. Such technologies are also widely used in India [39]. For example, the bioaccumulation rate in the roots and shoots of natural vegetation *Typha angustifolia* and *Echhornia crassipus* in the urban lake Lakshmi Taal, WW from the city of Jhansi in Central India was studied [40]. The possibility of using microalgae for the treatment of WW and natural waters from a wide range of pollutants, both organic (nitrates, sulfates) and inorganic (heavy metals), has been studied by many scientists, because of there a circular bioeconomy perspective [41]. *Chlorella sorokiniana* microalgae are widely used in the phytoremediation technologies [42]. In [43], a comprehensive work was carried out to study the treatment of urban WW using green algae *– Chlorella. sorokiniana* and *Scenedesmus obliquus*. In [44] authors were investigated the extraction of heavy metal ions — copper, nickel, cadmium, from drinking water by *Chlorella sorokiniana*, which was immobilized by extracting laminaria from algae. The processes of sorption of Pb, Cu, and Cd using green algae were studied in [45]. The microalgae *Chlorella sorokiniana, Chlorella vulgaris,* and *S. obliquus* are used as a sorbent for extracting drugs from pharmaceutical WW. Studies have been carried out to extract substances such as paracetamol, acetylsalicylic acid and diclofenac. Paracetamol is most effectively extracted by *Chlorella sorokiniana* strain [46].

Aquatic plants have an extensive root system that helps them accumulate pollutants in their roots and shoots [47, 48]. The cultivation of aquatic plants is time-consuming, which may limit the growing need for phytoremediation [49]. Nevertheless, this disadvantage is replaced by a number of advantages that this WW treatment technology possesses [50-52].

Various higher aquatic plants are used as biosorbents [53, 54]. Such plants must satisfy certain requirements: tolerance to toxicants and possible temperature changes, insect action, halophilicity, i.e. tolerance to increased salt concentrations, drought, possible stress factors, the presence of enzymes that ensure degradation toxicants, the ability to accumulate large quantities of inorganic toxicants in the intracellular space [55]. The most common plant-phytoremedants are eichhornia [56] and duckweed [57]. *Eichornia crassipes* (Water hyacinth) is a representative of higher aquatic vegetation. It is known that Eichornia is able to transform complex high-molecular and low-molecular compounds into simpler ones. Eichornia reaches the degree of water purification from compounds: nitrogen (up to 70%), phosphorus (up to 60%) and total organic carbon (up to 60%) [58, 59].

Duckweed is a free-floating aquatic plant that grows on the surface of the water. Duckweed belongs to the *Araceae* family (the *Lemnoideae* subfamily) and consists of five genera, in which at least 40 species have been identified [60]. Duckweed can survive at high pH (3.5 to 10.5) and temperatures from 7 to 35 ° C), so it is widely used in phytoremediation [61, 62]. Duckweed can eliminate a huge amount of various heavy metals, inorganic and organic pollutants, pesticides, nutrients come from agricultural WW, industrial and domestic WW, absorbs ammonia [49, 57, 63]. The nitrogen content in the cells of duckweed (trifoliate duckweed, *Lemna trisucula*, *Lemna minor*, etc.) may exceed its concentration in water by 2000 times, phosphorus - 7000, potassium - 5000 times [64, 65]. These properties of duckweed made it possible to use them for the purification of industrial and household water all over the world [66-68].

The main disadvantage of phytoremediation processes is the low speed and efficiency of purification [7, 69, 70]. To accelerate the processes of phytoremediation, various physical factors are used, the influence of which depends on wavelength, frequency of oscillations of electromagnetic waves, power and time of exposure [58, 59, 71]. For example, it is known that the effect of a magnetic field can manifest itself either as a stimulant or as a retardant for the development of cells and plant root systems [72-74] and, as a consequence, affect the phytosorption of heavy metals by plants during phytoremediation of polluted water bodies. It should be noted that a constant magnetic field (MF) has different effects on the growth, development and evolution of plants [75] – this was first noticed by the Russian scientist P.V. Savostin in 1930. In addition to magnetic fields [76], other influences were used to speed up the phytoremediation process, including: laser impact, ultraviolet and infrared radiation [58, 59]. It was shown that their effect on plants is associated with a change in the potential of cell membranes, an increase in their permeability and, as a consequence, an acceleration and completeness of cation absorption. Membranes, as a natural barrier, are the first to be exposed to physical factors, membranes are able to quickly respond to deviations in the conditions of existence. However, the changes that occur in the membranes entail a cascade of shifts in the metabolism of the entire cell. The permeability of membranes increases, depolarization of the membrane potential of the plasma membrane occurs, the pH (acidity) of the cytoplasm shifts to the acidic side, the activity of hydrogen ions H + increases [77, 78].

Copper (Cu) is one of the toxic heavy metals which widely used in modern industry, for example, in electronic chips, batteries, cell phones, etc. On the other hand, Cu is an essential element for human beings for healthy development, which quality is essential in the creation of hemoglobin in red platelets [7]. But, high dosages of copper can be to a great degree dangerous when it enters the human body through food, residue, and water and usage of copper-polluted water or foods can cause different dangerous diseases [79, 80]. Therefore, the permissible limit for the content of copper in drinking water according to the documents of the regulatory agencies of various countries is a fairly small value of 0.05 – 0.1 mg/L [81, 82]. For this reason, the results of research on methods for accelerating phytoremediation for purifying WW from copper ions presented in this paper are relevant in context of sustainable development and cleaner production. A lot of works are devoted to the influence of one physical factor, especially a magnetic field, on phytoremediation processes. The influence of the combined action of physical factors on the rate of extraction of pollutants is poorly studied. The objective of this research is to study the influence of the magnetic field strength, and the combined effects of magnetic and weak electric fields on the process of copper extraction using the higher aquatic plant *Lemna minor.* On this base also a specific technology has been proposed.

**2. Materials and Methods**

As the object of the phytoremediation study the highest aquatic duckweed plant *Lemna minor* was used. The influence of a magnetic field (MF) of different strengths (*H* = 0.0; 0.5; 1.0; 2.0; 4.0 kA/m) on copper extraction by *Lemna minor* duckweed was analyzed. The plants of the same maturity and weight - 20 g/L were planted in model solutions with a cation concentration of Cu^2 +^ 1 mg/L and subjected to a constant magnetic field in a specially designed setup (Fig. 1). CuSO_4_ was used to prepare a model solution. After 1, 2, 3, 4, 5, 24, 48, 120, 144, 168, 240 hours, the residual concentration of copper cation was measured. To study the combined effect of two factors, we used a magnetic field with a strength of H = 2 kA/m and a weak electric field with a current density of *j* = 80, 240, 480 μA/cm^2^. To create weak electric fields, we used the electrochemical system with volume 1 liter, where aluminum foil was used as the cathode, and a graphite rod was used as the anode. An electrochemical cell with duckweed in a CuSO_4_ solution (initial concentration of Cu^2 +^ was *C_i_* = 1 mg/L) was placed in a magnetic field and held for various time interval. After 1, 2, 3, 4, 5, 24, 48, 120, 144, 168, 240 hours, the residual concentration of copper cations was measured.

**Fig. 1.** Scheme of environment for experiments: 1 - DC power supply; 2 - magnetic coil; 3 - nickel-cadmium batteries; 4 - resistance store; 5 - voltammeter; 6 - aluminum electrode; 7 - duckweed; 8 - graphite electrode

To determine the final copper concentrations, the voltammetric method was used using the robotic complex with a “3 in 1” electrode. To extract copper from the spent biosorbent, an eluate was prepared using concentrated sulfuric acid. Then, the copper content in the eluate was determined by the potentiostatic method. The potentiostatic method was used to take a series of potentiostatic curves when extracting copper from the eluate at a constant potential. Copper was liberated at the cathode at potentials (*E* = 0.30; 0.32; 0.34 V) close to the copper release potential. According M. Faraday's laws of electrolysis, we can calculate the mass of the substance liberated at an electrode *m* in grams:

*m = Q M / (F z)*,	(1)

where: *Q* – is the total electric charge passed through the substance in coulombs, *F*= 96,485 C/mol is the Faraday constant, *M* – is the molar mass of the substance in g/mol, *z* - is the valency number of [ions](https://en.wikipedia.org/wiki/Ion) of the substance (electrons transferred per ion). In more simple applicable form for current case:

*m = q I t,*	(2)

where *q* = 1.185 g/(A h) is the electrochemical equivalent of Cu^2 +^, in the simple case of constant-current *I* (A h) electrolysis

Potentiostatic measurements were carried out at room temperature on a potentiostat with the results of the experiment recorded on a self-recording potentiometer. Graphite rods served as the working and auxiliary electrodes (the area of the auxiliary electrode was ~ 40 times larger than the working area). A normal silver chloride electrode in KCl solution was used as a reference electrode. During electro-chemical measurements, a sealed three-electrode cell with an electrolyte gate and separated cathode and anode spaces was used using Schott filters (Figure 2), which prevented the mixing of reaction products formed in the near-electrode layer.

**Fig. 2.** Three-electrode cell with separated Schott filters with cathode and anode spaces: *ae* – auxiliary, *we* – working, *nscc* – normal silver chloride comparison electrodes

Microstructural studies were performed on a SteREO Lumar luminescent stereo microscope. V12 of the German company Karl Zeiss with a smooth change of magnification zoom 1:12. and on a scanning electron microscope brand JSM-7001F, Jeol.

**3. Results and Discussions**

At the first stage, the influence of a constant magnetic field on phytoremediation processes was studied. When studying the effect of a magnetic field of different strengths (Figure 3), it was found that the removal of copper cations from sulfate solutions proceeds most efficiently in the initial period of 1-5 hours.

**Fig. 3.** Comparative results on the effect of MF of different strengths on the process of copper extraction of by *Lemna minor* duckweed: t- time (h), *Ci* - initial concentration Cu^2+^ mg/L = 1 mg/L, C_f_ Cu2 +- concentration of Cu^2+^, mg/L.

High metal removal rates are achieved up to ~ 5 days. The mechanism of bio-sorption by a plant of toxicants is carried out by the movement of dissolved substances through the cell membrane of plants. It can be seen in Figure 3 that, under the influence of a magnetic field with a strength of 2 kA/m, *Lemna minor* duckweed absorbs copper ions with greater speed and intensity than without magnetic field and with a field of 0.5;1.0; 4.0 kA/m. A 2 kA/m magnetic field exerts its effect as a stimulating factor. This fact is explained by an increase in the permeability of the plant cell membrane and stimulation of plant growth.

Therefore, at the next stage, we studied the effect of MF on the growth and reproduction of *Lemna minor* duckweed. It was found that the greatest growth and development of *Lemna minor* duckweed occurred without MF and when influenced to MF 2.0 kA/m (table 1). Fields with a strength of 0.5;1,0, 4 kA/m influenced on the duckweed depressingly.

**Table 1**

Dynamics of *Lemna minor* duckweed breeding with and without MF.

| Time, hours | The number of duckweed leaves in the sample, pieces | | | | |
| --- | --- | --- | --- | --- | --- |
|  | without MF | МF= 0,5 кА/м | МF= 1 кА/м | МF=2 кА/м | МF= 4 кА/м |
| 0 | 20 | 20 | 20 | 20 | 20 |
| 24 | 20 | 20 | 20 | 20 | 20 |
| 72 | 26 | 21 | 23 | 26 | 22 |
| 144 | 33 | 21 | 25 | 35 | 23 |
| 216 | 40 | 22 | 28 | 44 | 25 |
| 288 | 45 | 22 | 30 | 53 | 26 |
| 360 | 60 | 23 | 35 | 62 | 30 |
| 432 | 75 | 24 | 36 | 70 | 35 |
| 480 | 80 | 24 | 45 | 75 | 44 |

Upon desorption by the plants of the accumulated excess amount of copper, a change in their appearance was observed. Under the influence of a magnetic field of strength 0.5 kA/m on the second day, the duckweed changed its color from green to light green, it turned yellow on the 4th-5th day, necrosis was observed on the 10th day, and *Lemna minor* died. Under the influence of a magnetic field with a strength of 1.0 kA/m, a change in the color of the plant was also observed on the 2nd day from green to light green with a yellowish tint, on the 4th day the duckweed completely turned yellow, and on the 10th day the plant died. A somewhat better picture was observed under the influence of a magnetic field of 2.0 kA/m. On the second day, a discoloration of the leaves from green to light green with a yellow color was observed, on about the sixth day the leaves became brown-yellow, on the 15th day the duckweed completely turned brown and lost all signs of vitality, necrosis occurred. At *H* = 4.0 kA/m, *Lemna minor* duckweed actively degraded the structure of plant material, water penetrated into the phytomass, the plant color changed from bright green, in the initial state, to a brown color, during the period of inhibition. With further influence of the plant for 10 days, the plant died (Fig. 4).

At the next stage, the combined effect of magnetic fields and weak electric fields on the processes of copper extraction from sulfate solutions of duckweed *Lemna minor* was studied. Plants were placed in an electrochemical cell (with an aluminum cathode and a graphite anode located in a working solution of CuSO_4_) at given current densities *j* = 80, 240, 480 μA/cm^2^. The cell was placed in the facility creating a magnetic field with *H* = 2 kA/m as a value having the best effect of phytoremediation processes.

The obtained data on the influence of current density on the process of extracting copper duckweed made it possible to establish that the rate of water purification from copper cations increases with increasing influence time of the plant in solution (table 2). The minimum concentration and maximum rate of phytosorption of copper are achieved in the first hours of being duckweed in solution. The dependence is repeated regardless of the magnitude of the current density, at the same time, the extraction rate depends on these indicators.

| *а* | *b* | *c* | *d* |
| --- | --- | --- | --- |

Fig. 4. Photo of *Lemna minor* duckweed aged for 10 days: a, b - in natural conditions: a - (magnification x12); b - (x150); c, d - in a solution of CuSO_4_ with concentration 1 mg/L when influence ed to MF *H* = 4 kA/m: c - (x12); d - (x30)

**Table 2**

Concentration (*C*), purification efficiency *Pe*, %, extract velocity (*Ve*) of copper cations from a CuSO_4_ solution (1 mg /L Cu^2 +^) duckweed vs current densities (*j*) (MF strengths = 2 kA/m).

| Current density *j*, μA/cm^2^ | Time  *t*, h | Residual  concentration *C*, mg/L | Speed  extract *Ve*, mg/h | *Pe*, % |
| --- | --- | --- | --- | --- |
|  | *С_initial_* | 1,0 | - |  |
| 0 | 1 | 0,84+0,04 | 0,16 | 16 |
|  | 3 | 0,78+0,04 | 0,07 | 22 |
|  | 5 | 0,79+0,03 | 0,06 | 21 |
|  | 24 | 0,80+0,03 | 0,02 | 20 |
| 80 | 1 | 0,71+0,04 | 0,28 | 29 |
|  | 3 | 0,61+0,03 | 0,12 | 39 |
|  | 5 | 0,65+0,03 | 0,07 | 35 |
|  | 24 | 0,69+0,03 | 0,01 | 31 |
| 240 | 1 | 0,64+0,04 | 0,35 | 36 |
|  | 3 | 0,53+0,03 | 0,15 | 47 |
|  | 5 | 0,53+0,04 | 0,11 | 47 |
|  | 24 | 0,55+0,01 | 0,03 | 45 |
| 480 | 1 | 0,70+0,03 | 0,29 | 30 |
|  | 3 | 0,44+0,02 | 0,18 | 56 |
|  | 5 | 0,50+0,02 | 0,09 | 50 |
|  | 24 | 0,59+0,02 | 0,02 | 41 |

The lowest extraction velocity is achieved at a current density of 80 μA/cm^2^. The highest cleaning efficiency (up to 47%) is achieved when duckweed is kept in a CuSO_4_ solution at a current density of 240 μA/cm^2^. At a higher current (480 μA/cm^2^), the cleaning effect decreased, which is apparently due to the physical state of duckweed, which changed its external characteristics during biosorption. The plant lost its bright green color and became lighter. At the same time, we observed a more efficient extraction of copper by duckweed. After holding the plant for 5 hours, an excess concentration of copper in the solution was achieved. Subsequent copper recovery occurred at lower rates.

Our results on the effect of electric current are consistent with published data [82-86]. It is known that plant cell membranes have the ability to concentrate electric fields. When an additional external field is applied to the cells, the conductivity of cell membranes sharply increases. After moderate electric treatment (in our case, it is *j* = 80 μA/cm^2^), the cell conductivity, and, consequently, the biosorption process, gradually decreases. With more intense electrical treatment (*j* = 480 μA/cm^2^), an irreversible destruction of part of the cells occurs, which worsens the biosorption capacity of the membranes and, often, leads to electrical breakdown. The electric current *j* = 240 μA/cm2 is close to the biorhythm current, therefore, it contributes to an increase in cell permeability and has a beneficial effect on the biosorption of metal cations. Under the influence of an electric current, single electric pores appear in the cell membrane, which can change their sizes. The processes associated with changes in the cell membrane under the influence of an electric field are called electroporation [83]. During electroporation, a local restructuring of the structure occurs in the membrane, leading to the appearance of a through water channel through which micro and macro particles can travel at high speeds [87]. Large molecules are able to expand pores, which then slowly (~ 100 s) relax to the initial state.

The microstructural analysis of duckweed leaves subjected to physical stresses carried out using an electron scanning microscope (Fig. 5) made it possible to establish that the structure of their surface changes in the CuSO_4_ solution compared to the initial one. The cell structure under the influence of MF (*H* = 2 kA/m) and weak electric fields (240 μA/cm^2^), watering of plant tissues occurs.

| a | b |
| --- | --- |

**Fig. 5.** Microstructural studies of duckweed leaves *Lemna minor*: a - without influence in natural water; b - with the combined influence of magnetic field and electric field in a CuSO_4_ solution (x 2000).

The main problem of biosorption treatment is the regeneration and utilization of biosorbents. In the case of bioobjects, regeneration is not possible, therefore waste plants must be disposed of. Since there is no excessive accumulation of hazardous quantities of harmful substances in the plant phytomass, it can be used for paper and biofertilizers, processing for gas and liquid fuel after collection [31, 88]. Knowing the maximum permissible concentration (MPC) values of the content of heavy metals in animal feed (MPC Zn = 50 mg/kg; MPC Cd = 0.3 mg/kg; MPC Cu = 10 mg/kg; MPC Fe = 0.1 mg/kg), we can calculate the daily dose of spent phytosorbents as feed. But often waste plants contain toxic components that exceed the permissible value, in this case other methods of disposal are necessary.

At the same time, natural resources are used to obtain pure metals, and their extraction is harmful to the environment. The use of metals from recycled resources is economically beneficial and environmentally sound. Therefore, we studied the possibility of extracting copper from the spent phytomass of duckweed by the electrochemical (potentiostatic) method. This corresponds to the rules of the circular economy, where waste material (duckweed phytomass) becomes a raw material for a new product (pure copper).

Electrochemical extraction was carried out on duckweed, which was previously in a solution of copper sulfate under the combined influence of a magnetic field (intensity *H* = 2 kA/m) and weak electric fields with different current densities (*j* = 80, 240, 480 μA/cm^2^). The experimental results (Fig. 6) made it possible to establish that the highest copper content in the phytomass of the cassock plant was found during phytosorption of copper by the plant under the combined influence of MF *Н* = 2 kA/m and direct current density of 240 μA/cm^2^. This confirmed the presented data on the influence of weak electric fields on the enhancement of metal sorption by phytosorbents.

According to formulas (1) and (2), the mass of pure copper released on the electrode was calculated, determined by the potentiostatic method (PS) and a comparison was made with the mass of copper absorbed by the plant from the solution during phytoremediation, determined by the voltammetric method (VA) (table 3).

Fig. 6. Potentiostatic curves obtained when copper was isolated from the eluate of the spent phytomass of *Lemna minor* duckweed at *E* = 0.32 V. Duckweed was previously in a copper sulfate solution with Cu^2+^ 1 mg/L under the influence of MF = 2 kA/m, at various current densities *j,* μA/cm^2^.

**Table 3**

Comparative results of VA and PS methods for the determination of copper extracted from *Lemna minor* duckweed influenced to MF (H = 2 kA/m) and weak electric fields (j, μA/cm^2^) for 144 hours. The results are presented in mg per 100 g of phytosorbent for the following options: copper release potentials for PS method for 1 *E* = 0.30 V; for 2 *E* = 0.32 V; for 3 *E* = 0.34 V.

| Parameter, *j*, μA/cm^2^ | Research Methods | |
| --- | --- | --- |
|  | VA | PS |
| 0.00 1  2  3 | 0,233  0,215  0,239 | 0,232  0,213  0,230 |
| 80 1  2  3 | 0,325  0,271  0,294 | 0,273  0,266  0,283 |
| 240 1  2  3 | 0,381  0,385  0,372 | 0,320  0,312  0,311 |
| 480 1  2  3 | 0,241  0,240  0,234 | 0,238  0,238  0,230 |

Table 3 shows that the extraction of copper from the eluate of spent phytomass (PS) practically coincides with the mass of copper absorbed by the plant in the process of phytomass. The maximum metal release occurs at E = 0.32V. The developed electrochemical method of utilization of waste phytomass makes it possible to extract the metal almost completely. The recovered metal can be reused in the national economy, which will save natural resources. This corresponds to the principles of a circular economy of zero waste production, which is considered as a new business model for a more sustainable development of society. Much attention has been paid to such researches in recent years, because worldwide nowadays hot topics are environmentally safe use of resources and a green economy to improve the ecological situation in the world and increase the well-being of the population [89, 90].

**4. Technological recommendations**

To use phytoremediation processes on the basis of bioeconomics, technological recommendations have been developed and a scheme for WW treatment from copper using *Lemna minor* duckweed and physical influences has been proposed. The scheme of the proposed technology is presented in the figure 7.

**Fig. 7.** Schematic diagram of the phytoremediation of metals from WW: 1- WW averager; 2- pump for pumping averaged water into a biopond; 3- biopond populated by higher aquatic vegetation; 4- mesh pan for collecting and holding *Lemna minor* duckweed during the discharge of purified water; 5- installation of a constant magnetic field (can be combined with weak electric fields.); 6 - equipment for monitoring the residual content of copper cations in solution; 7- pump for pumping purified water; 8- lifting the waste mass by a pallet; 9- bath for the preparation of the eluate; 10- dispenser of sulfuric acid; 11 - metal electrolysis bath; 12- capacity for the collection of neutralized phytomass and its disposal

The results of the study made it possible to formulate the following technological recommendations:

1. *Lemna minor* duckweed is taken from natural conditions or grown in previously defended water at least until the ripening period (leaf size - 5 ... 6 mm). The plant should have a bright green color.
2. *Lemna minor* duckweed is transferred to the biopond with WW in an amount of 1.0 -1.5 kg/m^3^ of WW with an initial concentration, *Ci* of Cu^2 +^ 1 cation of 5 mg/L.
3. The further cleaning process is carried out under selected optimal conditions for influence to fields of various nature:

- Without physical effects, duckweed is kept in drains for 12-15 days to the required degree of treatment of WW.
- When influenced to MF with a field strength of *Н* = 2.0 kA/m, the plant is kept in WW for 8 days.
- When influenced to magnetic fields with a field strength of *H* = 2.0 kA/m together with a weak electric field *j* = 240 μA/cm^2^, the plant is kept in the WW for 6 days.

4. In the process of phytoremediation, the residual content of copper ions in the solution is controlled. The process is carried out to the maximum permissible concentration of copper ions in the MPC Cu^2 +^ solution = 0.1 mg/L - a toxicological indicator for water bodies.
5. Regular visual inspection of plants is done. When the color changes to pale green or brown (signs of cytoplasmolysis and necrosis), duckweed is removed from the biological pond and replaced with new plants.
6. From the spent biomass by electrochemical method pure copper is isolated, ready for further use.

This technology has several advantages: high cleaning efficiency, low cost and low energy consumption, versatility, environmental safety, ease of implementation, non-waste.

**5. Conclusions**

The objective of the research was to study the influence of the magnetic field strength, and the combined effects of magnetic and weak electric fields on the process of copper extraction using the higher aquatic plant *Lemna minor*. To accelerate the processes of extracting copper ions from wastewater using the higher aquatic plant duckweed *Lemna minor* the effect of influence of the magnetic field with various intensities (H, kA/m = 0.0; 0.5; 1.0; 2.0; 4.0) has been studied. This influence has been combined with influence of the weak electric field with a current density j, μA/cm^2^ = 80; 240 and 480. It was found that the removal of copper ions from sulfate solutions proceeds most effectively in the initial period of time (1-5 hours) and under the influence of a magnetic field with a strength of H = 2 kA/m. The research of the growth and development of duckweed fronds with and without influence to a magnetic field has been carried out. It was found that the greatest growth and development of Lemna minor duckweed occurred without influence and under the influence of a magnetic field with a strength of H = 2.0 kA/m. During these experiments, various changes in the external signs of the duckweed plants have been studied. It was shown that when duckweed is in sulfate solutions of copper, tissue necrosis occurs after 10 days.

The combined influence of a magnetic field (H = 2 kA/m) and weak electric fields has been studied. It was shown that the highest extraction rate was achieved when the duckweed is kept in a CuSO_4_ solution at a current density of j = 240 μA/cm^2^. After holding the plant for 5 h, the excess copper concentration was discharged into the solution. For the disposal and reuse of the spent phytomass, the electrochemical release of copper from the eluate of the spent phytomass was studied at potentials E = 0.3; 0.32; 0.34 V. The maximum metal release occurs at E = 0.32 V. It was shown that the maximum release of copper at the electrode occurs from the spent duckweed phytomass used in a magnetic field (Н = 2 kA/m) and a weak electric field j = 240 μA/cm^2^.

The advantages and disadvantages of the phytoremediation acceleration method proposed are analyzed on the example of the use of biosorbents for wastewater treatment. For the use of phytoremediation processes according to the principle of bioeconomics, technological recommendations have been developed. The scheme for purifying wastewater from copper with *Lemna minor* duckweed using physical influences to accelerate the process with the subsequent release of copper from the spent phytomass have been proposed. This scheme makes it possible to achieve waste-free technologies and a closed cycle of using duckweed for environmental purposes. This technology has a number of advantages: high purification efficiency, low cost and low energy consumption, versatility, environmental safety, ease of implementation, wastelessness. The implementation of this technological process in production or urban treatment facilities will ensure sustainable development of the economic sector, change the situation with natural resources, since the extracted metals from the spent phytosorbents will be used as secondary raw materials. The introduction of this technology will contribute to the development of the "green economy".

**Acknowledgment**

The research is partially funded by the Ministry of Science and Higher Education of the Russian Federation as part of World-class Research Center program: Advanced Digital Technologies (contract No. 075-15-2020-934 dated 17.11.2020)

**References**

1. Katyaini S, Barua A (2016) Water policy at science–policy interface–challenges and opportunities for India. Water Policy 18(2): 288-303. doi: 10.2166/wp.2015.086

2. Bolisetty S, Peydayesh M, Mezzenga R (2019) Sustainable technologies for water purification from heavy metals: review and analysis. Chem Soc Rev 48(2): 463-487. doi: 10.1039/c8cs00493e PMID: 30603760

3. WHO (2019) World Health Organization. Progress on household drinking water, sanitation and hygiene 2000-2017: special focus on inequalities. World Health Organization. – 140 p. https://www.who.int/water_sanitation_health/publications/jmp-report-2019/en/ (accessed 22 March 2021)

4. Klimes M, Michel D, Yaari E, Restiani P (2019) Water diplomacy: The intersect of science, policy and practice. J Hydrol 575: 1362–1370. doi: 10.1016/j.jhydrol.2019.02.049

5. Moghaddam VK, Changani F, Mohammadi A, Hadei M, Ashabi, R et al. (2017) Sustainable development of water resources based on wastewater reuse and upgrading of treatment plants: a review in the Middle East. Desalin Water Treat 65: 463-473. doi: 10.5004/dwt.2017.20383

6. Pattnaik P, Dangayach GS, Bhardwaj AK (2018) A review on the sustainability of textile industries wastewater with and without treatment methodologies. Rev Environ Health 33(2): 163-203. doi: 10.1515/reveh-2018-0013 PMID: 29858909

7. Vardhan KH, Kumar PS, Panda RC (2019) A review on heavy metal pollution, toxicity and remedial measures: Current trends and future perspectives. J Mol Liq 290: 111197. doi: 10.1016/j.molliq.2019.111197

8. McGeer JC, Brix KV, Skeaff JM, DeForest DK, Brigham SI et al. (2003) Inverse relationship between bioconcentration factor and exposure concentration for metals: implications for hazard assessment of metals in the aquatic environment. Environ Toxicol Chem 22(5): 1017-1037. doi: 10.1897/1551-5028(2003)022<1017:IRBBFA>2.0.CO;2 PMID: 12729211

9. Barakat MA (2011) New trends in removing heavy metals from industrial wastewater. Arab J Chem 4(4): 361-377. doi: 10.1016/j.arabjc.2010.07.019

10. Dubey S, Shri M, Gupta A, Rani V, Chakrabarty D (2018) Toxicity and detoxification of heavy metals during plant growth and metabolism. Environ Chem Lett 16(4): 1169-1192. doi: 10.1007/s10311-018-0741-8 PMID: 26940747

11. Chowdhury S, Mazumder MJ, Al-Attas O, Husain T (2016) Heavy metals in drinking water: occurrences, implications, and future needs in developing countries. Sci Total Env 569: 476-488. doi: 10.1016/j.scitotenv.2016.06.166 PMID: 27355520

12. Corda N, Kini MS (2020) Recent studies in adsorption of Pb (II), Zn (II) and Co (II) using conventional and modified materials: a review. Separation Science and Technology 55(15): 2679-2698. doi: 10.1080/01496395.2019.1652651

13. Khan FSA, Mubarak NM, Tan YH, Karri RR, Khalid M et al. (2020) Magnetic nanoparticles incorporation into different substrates for dyes and heavy metals removal ‑ A Review. Environ Sci Pollu. R 27(35): 43526-43541. doi: 10.1007/s11356-020-10482-z PMID: 32909134

14. Dhir B (2014) Potential of biological materials for removing heavy metals from wastewater. Environ Sci Pollut Research 21(3): 1614-1627. doi: 10.1007/s11356-013-2230-8 PMID: 24185905

15. Lin LC, Li JK, Juang RS (2008) Removal of Cu (II) and Ni (II) from aqueous solutions using batch and fixed-bed ion exchange processes. Desalination 225(1-3): 249-259. doi: 10.1016/j.desal.2007.03.017

16. Lesmana S.O, Febriana N, Soetaredjo FE, Sunarso J, Ismadji S (2009) Studies on potential applications of biomass for the separation of heavy metals from water and wastewater. Biochem Eng J 44(1): 19-41. doi: 10.1016/j.bej.2008.12.009

17. Fu F, Wang Q (2011) Removal of heavy metal ions from wastewaters: a review. J Environmen Manage 92(3): 407-418. doi: 10.1016/j.jenvman.2010.11.011 PMID: 21138785

18. Agarwal M, Singh K (2017) Heavy metal removal from wastewater using various adsorbents: a review. J Water Reuse Desal 7(4): 387-419. doi: 10.2166/wrd.2016.104

19. Collivignarelli MC, Abbà A, Bestetti M, Crotti BM, Miino MC (2019) Electrolytic recovery of nickel and copper from acid pickling solutions used to treat metal surfaces. Water Air Soil Poll 230(5): 1-13. doi: 10.1007/s11270-019-4158-1

20. Cao K, Zhi R, Zhang G (2020) Photosynthetic bacteria wastewater treatment with the production of value-added products: a review. Bioresource Technol 299: 122648. doi: 10.1016/j.biortech.2019.122648 PMID: 31889604

21. Carolin CF, Kumar PS, Saravanan A, Joshiba GJ, Naushad M (2017) Efficient techniques for the removal of toxic heavy metals from aquatic environment: A review. J Environ Chem Eng 5(3): 2782-2799. doi: 10.1016/j.jece.2017.05.029

22. Gautam RK, Mudhoo A, Lofrano G, Chattopadhyaya MC (2014) Biomass-derived biosorbents for metal ions sequestration: Adsorbent modification and activation methods and adsorbent regeneration. J Environ Chem 2(1): 239-259. doi: 10.1016/j.jece.2013.12.019

23. Krstić V, Urošević T, Pešovski B (2018) A review on adsorbents for treatment of water and wastewaters containing copper ions. Chem Eng Sci 192: 273-287. doi: 10.1016/j.ces.2018.07.022

24. Yi H, Li M, Huo X, Zeng G, Lai C et al. (2020) Recent development of advanced biotechnology for wastewater treatment. Crit Rev Biotechnol 40(1): 99-118. doi: 10.1080/07388551.2019.1682964 PMID: 31690134

25. Ashraf S, Ali Q, Zahir ZA, Ashraf S, Asghar HN (2019) Phytoremediation: Environmentally sustainable way for reclamation of heavy metal polluted soils. Ecotox Environ Safe 174: 714-727. doi: 10.1016/j.ecoenv.2019.02.068. PMID: 30878808

26. DalCorso G, Fasani E, Manara A, Visioli G, Furini A (2019) Heavy Metal Pollutions: State of the Art and Innovation in Phytoremediation. Int J Mol Sci 20(14): 3412. doi: 10.3390/ijms20143412. PMID: 31336773

27. Yan A, Wang Y, Tan SN, Yusof MLM, Ghosh S et al. (2020) Phytoremediation: A Promising Approach for Revegetation of Heavy Metal-Polluted Land. Front Plant Sci 11: 359. doi: 10.3389/fpls.2020.00359 PMID: 32425957

28. Pilon-Smits E (2005) Phytoremediation. Annu Rev Plant Biol 56: 15-39. doi: 10.1146/annurev.arplant.56.032604.144214 PMID: 15862088

29. Rezania S, Taib SM, Din MFM, Dahalan FA, Kamyab H (2016) Comprehensive review on phytotechnology: heavy metals removal by diverse aquatic plants species from wastewater. J Hazard Mater 318: 587-599. doi: 10.1016/j.jhazmat.2016.07.053 PMID: 27474848

30. Atia FA, Al-Ghouti MA, Al-Naimi F, Abu-Dieyeh M, Ahmed T, et al. (2019) Removal of toxic pollutants from produced water by phytoremediation: Applications and mechanistic study. J Water Process Eng 32:100990. doi: 10.1016/j.jwpe.2019.100990

31. Cui W, Cheng JJ (2015) Growing duckweed for biofuel production: a review. Plant biology 17: 16-23. doi: 10.1111/plb.12216 PMID: 24985498

32. Politaeva N, Smyatskaya Y, Tatarintseva EA (2020) Using Adsorption Material Based on the Residual Biomass of Chlorella Sorokiniana Microalgae for Wastewater Purification to Remove Heavy Metal Ions. Chem Petrol Eng 55(11-12): 907-912. doi: 10.1007/s10556-020-00712-z

33. Salt DE, Smith RD, Raskin I (1998) Phytoremediation. Annu Rev Plant Biol 49(1): 643-668. doi: 10.1146/annurev.arplant.49.1.643

34. Ali H, Khan E, Sajad MA (2013) Phytoremediation of heavy metals - concepts and applications. Chemosphere 91(7): 869-881. doi: 10.1016/j.chemosphere.2013.01.075 PMID: 23466085

35. Mulkeen CJ, Williams CD, Gormally MJ, Healy MG (2017) Seasonal patterns of metals and nutrients in Phragmites australis (Cav.) Trin. ex Steudel in a constructed wetland in the west of Ireland. Ecol Eng 107: 192-197. doi: 10.1016/j.ecoleng.2017.07.007

36. Lesage E, Rousseau DPL, Meers E, Van de Moortel AMK, Du Laing G et al. (2007) Accumulation of metals in the sediment and reed biomass of a combined constructed wetland treating domestic wastewater. Water Air Soil Pollut 183(1-4): 253-264. doi: 10.1007/s11270-007-9374-4.

37. Yu R, Wu Q, Lu X (2012) Constructed wetland in a compact rural domestic wastewater treatment system for nutrient removal. Environ Eng Sci 29(8): 751-757. doi: 10.1089/ees.2011.0209

38. Su F, Li Z, Li Y, Xu L, Li Y et al. (2019) Removal of total nitrogen and phosphorus using single or combinations of aquatic plants. Int J Env Res Pub He 16(23): 4663. doi: 10.3390/ijerph16234663 PMID: 31771100

39. Nayak S, Kale P (2020). A review of chromite mining in Sukinda Valley of India: impact and potential remediation measures. Int J Phytoremediat 22(8): 804-818. doi: 10.1080/15226514.2020.1717432 PMID: 32028787

40. Pandey SK, Upadhyay RK, Gupta VK, Worku K, Lamba D (2019) Phytoremediation Potential of Macrophytes of Urban Waterbodies in Central India. J Health Pollut 9(24): 191206. doi: 10.5696/2156-9614-9.24.191206 PMID: 31893167

41. Nagarajan D, Lee DJ, Chen CY, Chang JS (2020) Resource recovery from wastewaters using microalgae-based approaches: a circular bioeconomy perspective. Bioresource Technol 302: 122817. doi: 10.1016/j.biortech.2020.122817 PMID: 32007309

42. Jacob JM, Karthik C, Saratale RG, Kumar SS, Prabakar D et al. (2018) Biological approaches to tackle heavy metal pollution: a survey of literature. J Environ Manage 217: 56-70. doi: 10.1016/j.jenvman.2018.03.077 PMID: 29597108

43. Gupta SK, Ansari FA, Shriwastav A, Sahoo NK, Rawat I et al. (2016) Dual role of Chlorella sorokiniana and Scenedesmus obliquus for comprehensive wastewater treatment and biomass production for bio-fuels. J Clean Prod 115: 255-264. doi: 10.1016/j.jclepro.2015.12.040

44. Petrovič A, Simonič M (2016) Removal of heavy metal ions from drinking water by alginate-immobilised Chlorella sorokiniana. Int J Environ Sci Te 13(7): 1761-1780. doi: 10.1007/s13762-016-1015-2

45. Liang S, Kang Y, Zeng L, Qin Y, Wang L et al. (2017) How Chlorella sorokiniana and its high tolerance to Pb might be a potential Pb biosorbent. Pol J Environ Stud 26(3): 1139-1146. doi: 10.15244/pjoes/67532

46. Escapa C, Coimbra RN, Paniagua S, García AI, Otero M (2017) Paracetamol and salicylic acid removal from contaminated water by microalgae. J Environ Manage 203: 799-806. doi: 10.1016/j.jenvman.2016.06.051 PMID: 27421699

47. Mays P, Edwards G (2001) Comparison of heavy metal accumulation in a natural wetland and constructed wetlands receiving acid mine drainage. Ecol Eng 16: 487–500. doi: 10.1016/S0925-8574(00)00112-9

48. Stoltz E, Greger M (2002) Accumulation properties of As, Cd, Cu, Pb and Zn by four wetland plant species growing on submerged mine tailings. Environ Exp Bot 47: 271–280. doi: 10.1016/S0098-8472(02)00002-3

49. de Souza TD, Borges AC, Braga AF, Veloso RW, de Matos AT (2019) Phytoremediation of arsenic-contaminated water by Lemna Valdiviana: An optimization study. Chemosphere. 234: 402-408. doi: 10.1016/j.chemosphere.2019.06.004 PMID: 31228843

50. Valipour A, Hamnabard N, Woo KS, Ahn YH (2014) Performance of high-rate constructed phytoremediation process with attached growth for domestic wastewater treatment: Effect of high TDS and Cu. J Environ Manage 145: 1-8. doi: 10.1016/j.jenvman.2014.06.009 PMID: 24981281

51. Thijs S, Sillen W, Rineau F, Weyens N, Vangronsveld J (2016) Towards an enhanced understanding of plant–microbiome interactions to improve phytoremediation: engineering the metaorganism. Front Microbiol 7: 341. doi: 10.3389/fmicb.2016.00341 PMID: 27014254

52. Koźmińska A, Wiszniewska A, Hanus-Fajerska E, Muszyńska E (2018) Recent strategies of increasing metal tolerance and phytoremediation potential using genetic transformation of plants. Plant Biotechnol Rep 12: 1–14. doi: 10.1007/s11816-017-0467-2 PMID: 29503668

53. Park S, Kang D, Kim Y, Lee SM, Chung Y et al. (2011) Biosorption and growth inhibition of wetland plants in water contaminated with a mixture of arsenic and heavy metals. Eng Life Sci 11(1): 84-93. doi: 10.1002/elsc.201000024

54. Vanhoudt N, Vandenhove H, Leys N, Janssen P (2018) Potential of higher plants, algae, and cyanobacteria for remediation of radioactively contaminated waters. Chemosphere 207: 239-254. doi: 10.1016/j.chemosphere.2018.05.034 PMID: 29803156

55. Mishra S, Maiti A (2017) The efficiency of Eichhornia crassipes in the removal of organic and inorganic pollutants from wastewater: a review. Environ Sci Pollut R 24(9): 7921-7937. doi: 10.1007/s11356-016-8357-7 S PMID: 28092006

56. Li F, Shen K, Long X, Wen J, Xie X et al. (2016) Preparation and characterization of biochars from Eichornia crassipes for cadmium removal in aqueous solutions. PLoS ONE 11(2): e0148132. doi: 10.1371/journal.pone.0148132 PMID: 26882239

57. Ekperusi AO, Sikoki FD, Nwachukwu EO (2019) Application of common duckweed (Lemna minor) in phytoremediation of chemicals in the environment: State and future perspective. Chemosphere. 223: 285-309. doi: 10.1016/j.chemosphere.2019.02.025 PMID: 30784736

58. Politaeva NA, Olshanskaya LN, Ovchinnikov FA, Sverguzova SV (2019) Influence of IR radiation on the process of phytoremediation of copper by eichhornia. IOP C Ser Earth Env 288(1): 012057. [doi: 10.1088/1755-1315/288/1/012057](http://dx.doi.org/10.1088/1755-1315/288/1/012057)

59. Politayeva NA, Olshanskaya LN, Trukhina EV, Timkovskii AL, Lezhnev EI et al. (2019) Influence of laser radiation on processes of electrochemical phytoremediation of copper from waste water by eichhornia. IOP C Ser Earth Env 337(1): 012075. doi: 10.1088/1755-1315/337/1/012075

60. Ziegler P, Sree KS, Appenroth KJ (2017) The uses of duckweed in relation to water remediation. Desalin Water Trea 63: 327-342. doi: 10.5004/dwt.2017.0479

61. Basiglini E, Pintore M, Forni C (2018) Effects of treated industrial wastewaters and temperatures on growth and enzymatic activities of duckweed (Lemna minor L.). Ecotoxi Environ Safe 153:54-59. doi: org/10.1016/j.ecoenv.2018.01.053 PMID: 29407738

62. Ali S, Abbas Z, Rizwan M, Zaheer IE, Yavaş İ, et al. (2020) Application of floating aquatic plants in phytoremediation of heavy metals polluted water: a review. Sustainability 12(5): 1927. doi: 10.3390/su12051927

63. Mohedano RA, Costa RH, Tavares FA, Belli Filho P (2012) High nutrient removal rate from swine wastes and protein biomass production by full-scale duckweed ponds. Bioresour Technol 112: 98–104. doi: 10.1016/j.biortech.2012.02.083 PMID: 22425517

64. Daud M, Ali S, Abbas Z, Zaheer IE, Riaz, M.A. et al. (2018) Potential of Duckweed (Lemna minor) for the Phytoremediation of Landfill Leachate. J Chem 2018: 3951540. doi: 10.1155/2018/3951540

64. Sallah-Ud-Din R, Farid M, Saeed R, Ali S, Rizwan M et al. (2017) Citric acid enhanced the antioxidant defense system and chromium uptake by Lemna minor L. grown in hydroponics under Cr stress. Environ Sci Pollut Res 24: 17669–17678. doi: 10.1007/s11356-017-9290-0 PMID: 28600794

65. Zhang K, Chen YP, Zhang TT, Zhao Y, Shen Y et al. (2014) The logistic growth of duckweed (Lemna minor) and kinetics of ammonium uptake. Environ Technol 35: 562–567. doi: 10.1080/09593330.2013.837937 PMID: 24645435

66. Smith MD, Moelyowati I (2001) Duckweed based wastewater treatment (DWWT): design guidelines for hot climates. Water Sci Technol 43(11): 291-299. doi: 10.2166/wst.2001.0694 PMID: 11443975

67. Hettiarachchi M, McAlpine C, Morrison TH (2014) Governing the urban wetlands: a multiple case-study of policy, institutions and reference points. Environ Conserv 41(3): 276-289. doi: 10.1017/S0376892913000519

68. Gorito AM, Ribeiro AR, Almeida CMR, Silva AM (2017) A review on the application of constructed wetlands for the removal of priority substances and contaminants of emerging concern listed in recently launched EU legislation. Environ Pollut 227: 428–443. doi: 10.1016/j.envpol.2017.04.060 PMID: 28486186

69. Gałczyńska M, Mańkowska N, Milke J, Buśko M (2019) Possibilities and limitations of using Lemna minor, Hydrocharis morsus-ranae and Ceratophyllum demersum in removing metals with contaminated water. J Water Land Dev 40(1): 161-172. doi: 10.2478/jwld-2019-0018

70. Rahman Z, Singh VP (2020) Bioremediation of toxic heavy metals (THMs) contaminated sites: concepts, applications and challenges. Environ Sci Pollut R 27(22): 27563-27581. doi: 10.1007/s11356-020-08903-0 PMID: 32418096

71. Luo J, He W, Qi S, Wu J, Gu XS (2020) A novel phytoremediation method assisted by magnetized water to decontaminate soil Cd based on harvesting senescent and dead leaves of Festuca arundinacea. J Hazard Mater 383: 121115. doi: 10.1016/j.jhazmat.2019.121115 PMID: 31525682

72. Maffei ME (2014) Magnetic field effects on plant growth, development, and evolution. Front Plant Sci 5: 445. doi: 10.3389/fpls.2014.00445 PMID: 25237317

73. Olshanskaya LN, Titorenko OV, Eremeeva YA (2015) Influence of a Constant Magnetic Field and Ultraviolet Radiation on Growth of Higher Plants and Phytoremediation of Soil from Oil Products. Chem Petrol Eng 51(5): 361-365. doi: 10.1007/s10556-015-0052-x

74. Luo J, He W, Xing X, Wu J, Gu XS (2019) The phytoremediation efficiency of Eucalyptus globulus treated by static magnetic fields before sowing. Chemosphere 226: 891-897. doi: 10.1016/j.chemosphere.2019.03.192 PMID: 31509918

75. Da Silva JAT, Dobránszki J (2016) Magnetic fields: how is plant growth and development impacted? Protoplasma 253(2): 231-248. doi: 10.1007/s00709-015-0820-7 PMID: 25952081

76. Luo J, He W, Yang D, Wu J, Gu XS (2019) Magnetic field enhance decontamination efficiency of Noccaea caerulescens and reduce leaching of non-hyperaccumulated metals. J Hazard Mater 368: 141-148. doi: 10.1016/j.jhazmat.2019.01.046 PMID: 30669038

77. Levin M (2012) Molecular bioelectricity in developmental biology: new tools and recent discoveries: control of cell behavior and pattern formation by transmembrane potential gradients. Bioessays 34(3): 205-217. doi: 10.1002/bies.201100136 PMID: 22237730

78. Bassetto VC, Russell AE, Kubota LT, Bartlett PN (2014) Preparation of copper sphere segment void templates for electrochemical SERS and their use to study the interaction of amino acids with copper under potentiostatic control. Electrochim Acta 144: 400-405. doi: 10.1016/j.electacta.2014.08.066

79. Brewer GJ (2010) Risks of copper and iron toxicity during aging in humans. Chem Res Toxicol 23(2): 319-326. doi: 10.1021/tx900338d

80. Huat TJ, Camats-Perna J, Newcombe EA, Valmas N, Kitazawa M et al. (2019) Metal toxicity links to Alzheimer's disease and neuroinflammation. J Mol Biol 431(9): 1843-1868. doi: 10.1016/j.jmb.2019.01.018 PMID: 30664867

81. WHO (2004) Copper in Drinking-water, World Health Organization. Background document for development of WHO guidelines for drinking-water quality https://www.who.int/water_sanitation_health/publications/copper/en/ (accessed 22 March 2021)

82. Esser AT, Smith KC, Gowrishankar TR, Vasilkoski Z, Weaver JC (2010) Mechanisms for the intracellular manipulation of organelles by conventional electroporation. Biophys J 98(11): 2506-2514. doi: 10.1016/j.bpj.2010.02.035 PMID: 20513394

83. Weaver JC, Chizmadzhev YA (1996) Theory of electroporation: a review. Bioelectroch Bioenerg 41(2): 135-160. doi: 10.1016/S0302-4598(96)05062-3

84. Kikuchi T, Tanaka S (2012) Biological removal and recovery of toxic heavy metals in water environment. Crit Rev Env Sci Tec 42(10): 1007-1057. doi: 10.1080/10643389.2011.651343 PMID: 16427277

85. Kotnik T, Frey W, Sack M, Meglič SH, Peterka M et al. (2015) Electroporation-based applications in biotechnology. Trends Biotechnol 33(8): 480-488. doi: 10.1016/j.tibtech.2015.06.002 PMID: 26116227

86. Kar S, Loganathan M, Dey K, Shinde P, Chang HY et al. (2018) Single-cell electroporation: current trends, applications and future prospects. J Micromech Microeng 28(12): 123002. doi: 10.1088/1361-6439/aae5ae

87. Son RS, Gowrishankar TR, Smith KC, Weaver JC (2015) Modeling a conventional electroporation pulse train: decreased pore number, cumulative calcium transport and an example of electrosensitization. IEEE T on Bio-Med Eng 63(3): 571-580. doi: 10.1109/TBME.2015.2466234 PMID: 26302502

88. Bert V, Neub S, Zdanevitch I, Friesl-Hanl W, Collet S, et al. (2017) How to manage plant biomass originated from phytotechnologies? Gathering perceptions from end-users. Int J Phytoremediat 19(10):947-954. doi: 10.1080/15226514.2017.1303814 PMID: 28323452

89. Ghisellini P, Cialani C, Ulgiati S (2016) A review on circular economy: the expected transition to a balanced interplay of environmental and economic systems. J Clean Prod 114: 11-32. doi: 10.1016/j.jclepro.2015.09.007

90. Figge F, Thorpe AS (2019) The symbiotic rebound effect in the circular economy. Ecol Econ 163: 61-69. doi: 10.1016/j.ecolecon.2019.04.028
