# Supplementary material for: Magnetic and electric field accelerate Phytoextraction of copper Lemna minor duckweed
Source: PLoS One. 2021 Aug 4;16(8):e0255512. doi: 10.1371/journal.pone.0255512 (PMC8336833; doi:10.1371/journal.pone.0255512)
Supplement: Attachment — Submitted filename: Response to Reviewers.docx [file pone.0255512.s002.docx]

**Journal Requirements:**

**When submitting your revision, we need you to address these additional requirements.**

1. Please ensure that your manuscript meets PLOS ONE's style requirements, including those for file naming. The PLOS ONE style templates can be found at

https://journals.plos.org/plosone/s/file?id=wjVg/PLOSOne_formatting_sample_main_body.pdf and

[https://journals.plos.org/plosone/s/file?id=ba62/PLOSOne_formatting_sample_title_authors_affiliations.pdf](about:blank)

Response:

We have edited our manuscript, and we hope now our manuscript meets PLOS ONE's style requirements, including those for file naming.

2.Thank you for stating the following in the Acknowledgments Section of your manuscript:

"The research is partially funded by the Ministry of Science and Higher Education of the
Russian Federation as part of World-class Research Center program: Advanced Digital
Technologies (contract No. 075-15-2020-934 dated 17.11.2020)"

We note that you have provided funding information that is not currently declared in your Funding Statement. However, funding information should not appear in the Acknowledgments section or other areas of your manuscript. We will only publish funding information present in the Funding Statement section of the online submission form.

Please remove any funding-related text from the manuscript and let us know how you would like to update your Funding Statement. Currently, your Funding Statement reads as follows:

 "The funders had no role in study design, data collection and analysis, decision to publish, or preparation of the manuscript."

Please include your amended statements within your cover letter; we will change the online submission form on your behalf.

Response:

We have removed any funding-related text from the manuscript

We would like to update our Funding Statement as following: "The research is partially funded by the Ministry of Science and Higher Education of the Russian Federation as part of World-class Research Center program: Advanced Digital Technologies (contract No. 075-15-2020-934 dated 17.11.2020)"

We also have included amended statement within our cover letter.

**Reviewers' comments:**

**Reviewer's Responses to Questions**

**Comments to the Author**

1. Is the manuscript technically sound, and do the data support the conclusions?

The manuscript must describe a technically sound piece of scientific research with data that supports the conclusions. Experiments must have been conducted rigorously, with appropriate controls, replication, and sample sizes. The conclusions must be drawn appropriately based on the data presented.

Reviewer #1: Partly

Reviewer #2: Yes

Response:

We have partially improved the manuscript from this point of view

2. Has the statistical analysis been performed appropriately and rigorously?

Reviewer #1: No

Reviewer #2: No

Response:

We have improved the manuscript from this point of view. We have improved Table 1 and Table 3. It was found that critical value of Student's criterion = 95% for our experimental data.

3. Have the authors made all data underlying the findings in their manuscript fully available?

The [PLOS Data policy](http://www.plosone.org/static/policies.action#sharing) requires authors to make all data underlying the findings described in their manuscript fully available without restriction, with rare exception (please refer to the Data Availability Statement in the manuscript PDF file). The data should be provided as part of the manuscript or its supporting information, or deposited to a public repository. For example, in addition to summary statistics, the data points behind means, medians and variance measures should be available. If there are restrictions on publicly sharing data—e.g. participant privacy or use of data from a third party—those must be specified.

Reviewer #1: Yes

Reviewer #2: Yes

Response:

According reviewer answers, there is nothing to improve.

4. Is the manuscript presented in an intelligible fashion and written in standard English?

PLOS ONE does not copyedit accepted manuscripts, so the language in submitted articles must be clear, correct, and unambiguous. Any typographical or grammatical errors should be corrected at revision, so please note any specific errors here.

Reviewer #1: Yes

Reviewer #2: No

Response:

We have partially improved the manuscript from this point of view. Small corrections are highlighted by red color of the text and otherwise corrections are highlighted using yellow background.

5. Review Comments to the Author

Please use the space provided to explain your answers to the questions above. You may also include additional comments for the author, including concerns about dual publication, research ethics, or publication ethics. (Please upload your review as an attachment if it exceeds 20,000 characters)

**Reviewer #1:** The abstract section is not appropriate. The authors have not included the results of the study. Presently, the Abstract section is just an introduction to the study. I would advise organizing it the following way.

1: introductory sentences (1-2 sentences); 2: methodology (1-2 sentences); 3: description of the numerical results must be the main body of the Abstract (5-10 sentences); 4: conclusion (1-2 sentences. )

Response:

We have completely rewritten the abstract according recommendation

It's 'Keywords' and not the 'Key words'.

Arrange your keywords alphabetically

Response:

We have rewritten the keywords

The authors didn't provide any references for the adopted experimental protocols/procedures. Moreover, the provided details are not enough to reproduce the study. Please, include the complete details of the methods with proper reference citations.

There is no statistical analysis for Table 1.

Response:

We have added some additional explanation in the text

I don't know what has happened to Figure 1. There is just a Table and no figure(s).

Response:

All figures for the manuscript have been improved. All figures have been checked in Preflight Analysis and Conversion Engine (PACE) digital diagnostic tool, <https://pacev2.apexcovantage.com/>.

Statistical analysis for the data presented in Table 2 is not sufficient. Please, compare it for significant differences.

Response:

Table 2 have been edited

No figure in Figure 5.

Response:

All figures for the manuscript have been improved. All figures have been checked in Preflight Analysis and Conversion Engine (PACE) digital diagnostic tool, <https://pacev2.apexcovantage.com/>.

Reduce the conclusion section up to 5-8 lines.

Response:

We have completely rewritten the conclusions section

Replace the references older than 10 years. You can include a reference older than 10 years if it is imperative to mention it due to exceptional circumstances.

Response:

We have replaced old references (see yellow background in References section)

Be selective and representative while citing the studies. A regular article should have 40-50 citations.

Response:

We have reduced the number of references

Please consider the following if you submit the revised version of the study to the journal or elsewhere.

1: must include line numbers

Yes, we have included line numbers

2: improve the quality of the pictures to highlight the main object/finding in it

All figures for the manuscript have been improved. All figures have been checked in Preflight Analysis and Conversion Engine (PACE) digital diagnostic tool, <https://pacev2.apexcovantage.com/>.

3: arrange your figure to develop pictorial plates. (please, see the published papers to see how the pictures are arranged into pictorial plates)

All figures for the manuscript have been improved. All figures have been checked in Preflight Analysis and Conversion Engine (PACE) digital diagnostic tool, <https://pacev2.apexcovantage.com/>.

**Reviewer #2:** The research presented in the manuscript is of significant quality. However, its presentation can be further improved by analyzing the data statistically. Moreover, English language editing is recommended. Few suggestions and comments are given as track changes.

Response:

We have partially improved the manuscript from this point of view. Small corrections are highlighted by red color of the text and otherwise corrections are highlighted using yellow background.

6. PLOS authors have the option to publish the peer review history of their article ([what does this mean?](https://journals.plos.org/plosone/s/editorial-and-peer-review-process#loc-peer-review-history)). If published, this will include your full peer review and any attached files.


If you choose “no”, your identity will remain anonymous but your review may still be made public.


**Do you want your identity to be public for this peer review?** For information about this choice, including consent withdrawal, please see our [Privacy Policy](https://www.plos.org/privacy-policy).

Reviewer #1: No

Reviewer #2: **Yes:** Muhammad Baqir Hussain

[NOTE: If reviewer comments were submitted as an attachment file, they will be attached to this email and accessible via the submission site. Please log into your account, locate the manuscript record, and check for the action link "View Attachments". If this link does not appear, there are no attachment files.]

These comments do not require a response.
